# Endocrine Evaluation in POEMS Syndrome: A Cohort Study

**DOI:** 10.3389/fendo.2020.536241

**Published:** 2020-10-27

**Authors:** Hongbo Yang, Hao Zhao, Xuemin Gao, Xufei Huang, Xinxin Cao, Daobin Zhou, Weibo Xia, Jian Li

**Affiliations:** ^1^Department of Endocrinology, Key laboratory of Endocrinology of National Health Commission, The Translational Medicine Center of PUMCH, Beijing, China; ^2^Department of Hematology, Peking Union Medical College Hospital, Chinese Academy of Medical Sciences and Peking Union Medical College, Beijing, China

**Keywords:** POEMS syndrome, endocrinopathy, thyroid response, risk stratification, overall survival

## Abstract

Endocrinopathy is an important characteristic of POEMS (polyneuropathy, organomegaly, endocrinopathy, monoclonal gammopathy, and skin changes) syndrome. However, endocrine responses to different regimens were unknown so far. Here we investigated endocrine characteristics in 383 patients with newly diagnosed POEMS syndrome and thyroid responses 1 year after treatment with autologous peripheral stem cell transplantation, melphalan plus dexamethasone, or lenalidomide plus dexamethasone. Overt hypothyroidism and subclinical hypothyroidism were noted in 20.6% (79/383) and 36.0% (138/383) of patients. Adrenal insufficiency was noted in 13.6% (43/316) of patients. Hyperprolactinemia was noted in 62.7% (207/330) of patients. Hypogonadism was noted in 48.0% (60/125) of female and 22.6% (51/226) of male patients. Thyroid function was significantly related with baseline risk stratification (*p* < 0.001) and significantly improved regardless of initial regimens. Patients with baseline hypothyroidism had a significant inferior progression-free survival (PFS) (*p* = 0.028) and overall survival (OS) (*p* = 0.006). Three-year PFS in patients with and without baseline hypothyroidism were 68.9 vs. 82.5%, respectively. Three-year OS rates in patients with and without baseline hypothyroidism were 82.8 vs. 92.8%, respectively. In summary, hypothyroidism, hyperprolactinemia, and hypogonadism are common endocrinopathies in POEMS syndrome. Thyroid function significantly improved regardless of the initial regimens. Thyroid function parallels with baseline risk stratification, and patients with baseline hypothyroidism have significantly inferior OS and PFS.

## Introduction

Polyneuropathy, organomegaly, endocrinopathy, monoclonal gammopathy, and skin changes (POEMS) syndrome is an important paraneoplastic syndrome due to underlying plasma cell disorder (1). Dominant clinical characteristics are typically neuropathy, endocrine disturbances, volume overload, and elevated serum vascular endothelial growth factor (VEGF) levels. There are no published randomized clinical trials among POEMS syndrome so far with combined therapy. The main systemic treatment strategy is targeting at plasma cell clones, including autologous stem cell transplantation (ASCT), melphalan-based therapy, and novel agent–based therapy (lenalidomide, etc.) (2). Monoclonal protein and VEGF levels are used to monitor disease activity (3).

Endocrinopathy is an enigma in POEMS syndrome. In POEMS case series from Mayo Clinic, 84% of the 64 patients had a recognized endocrinopathy, with hypogonadism and hypothyroidism as the most common disturbances, followed by abnormal glucose metabolism and adrenal insufficiency. Fifty-four percent had evidence of multiple endocrinopathies in the four major endocrine axes (4). In a recent report from Europe, endocrinopathy was found in 63% of patients at diagnosis and in 92% of patients during follow-up, mostly hypogonadism and hypothyroidism (5). In our retrospective study of 99 patients with newly diagnosed POEMS syndrome (6), hypothyroidism (67%) was the most common endocrinopathy. Impotence (89%), gynecomastia (12%), and low testosterone levels (56%) were common findings in men. Improvement of thyroid function and sexual function in POEMS patients after combination therapy of lenalidomide and dexamethasone was also found in our previous studies (7, 8). Whether the improvement of thyroid function and sexual function is regimen specific or not is underinvestigated.

In this study, we retrospectively analyzed the endocrine characteristics and responses of thyroid function to different regimens in a large cohort of patients with POEMS syndrome in China.

## Materials and Methods

### Patients and Regimens

The medical records of 383 consecutive cases with POEMS syndrome were retrospectively analyzed. Diagnostic criteria were defined by Dispenzieri (1), with two mandatory criteria (polyneuropathy and monoclonal plasma cell proliferating disorder), at least one major criterion (sclerotic bone lesion, Castleman, disease or VEGF elevation), and one minor criterion (organomegaly, edema, endocrinopathy, skin change, papillary edema, or thrombocytosis). All patients were admitted to Peking Union Medical College Hospital between January 2000 and July 2018. Approval from the institutional review board of Peking Union Medical College Hospital was obtained for this study. All data were deidentified before analysis.

**Figure 1:**
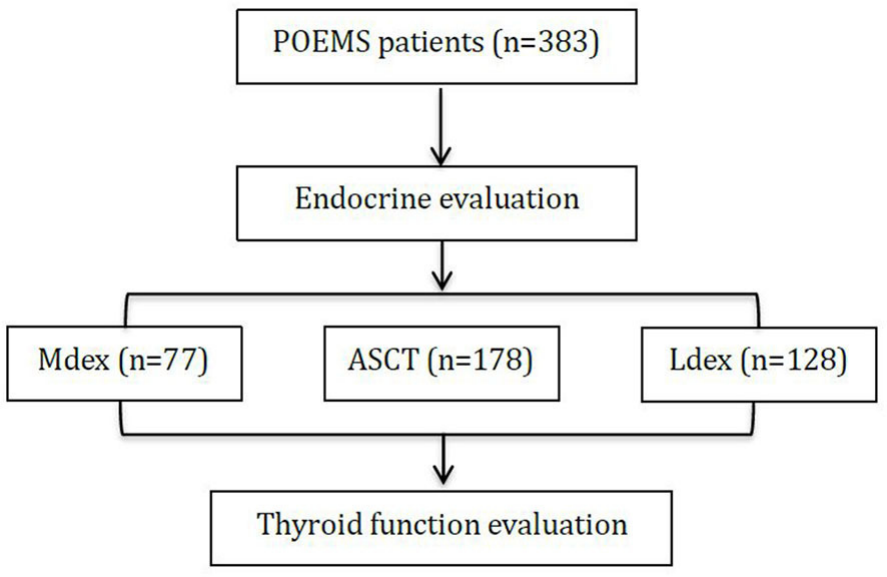
The flowchart of the study. Combination therapy of melphalan and dexamethasone regimen (MDex), autologous peripheral stem cell transplantation (ASCT), and low-dose lenalidomide plus dexamethasone regimen (LDex).

The treatment algorithm is mainly based on the extent of the plasma cell infiltration. Patients with disseminated disease as defined by bone marrow involvement or over two bone lesions would receive systematic treatment as recommended by Dispenzieri (9). Among our patients (Figure 1), 178 were treated by autologous peripheral stem cell transplantation. Patients eligible for ASCT were younger than 65 years, without serious systemic disease or organ dysfunction, severe pulmonary hypertension, severe capillary leak syndrome (hypotension and/or refractory ascites), and active infection and with successful collection of adequate peripheral blood stem cells, as we described in a previous article (10). Seventy-seven patients received combination therapy of melphalan and dexamethasone regimen (MDex) as described previously (2). MDex consisted of oral melphalan (10 mg/m^2^ body surface area) plus oral dexamethasone (40 mg/d) on days 1 to 4 every 28-day cycle. One hundred twenty-eight patients received low-dose lenalidomide plus dexamethasone regimen (LDex) as described previously (11). LDex consisted of oral lenalidomide (10–25 mg daily) for 21 days of a 28-day cycle, plus oral dexamethasone (40 mg once per week). Aspirin (100 mg daily) was prescribed for prophylaxis for thrombosis. Patients were followed for a median of 25 months after the last cycle of treatment (range, 1–227 months).

### Definition of Variables

As reported previously (7), adrenocorticotropic hormone (ACTH) deficiency was defined as a serum cortisol level of <3.0 pg/mL at 8 a.m. and an inappropriate low or normal ACTH level (<46 pg/mL). Euthyroidism was defined as normal thyroxine (T4) and thyrotropin (TSH) levels. Subclinical hypothyroidism was defined as elevated TSH and normal serum free thyroxine (FT4) levels. Overt hypothyroidism was defined as reduced free or total T4 and elevated TSH levels. Hypogonadotropic hypogonadism was defined as low serum testosterone/estradiol levels and a low luteotropic hormone level. Hyperprolactinemia was defined with a serum prolactin levels higher than the upper limit of normal range.

### Risk Stratification

Risk stratification system was developed previously (12). Briefly, four baseline clinical variables including age (>50 years), pulmonary hypertension, pleural effusion, and estimated glomerular filtration rate <30 mL/min per 1.73 m^2^ were associated with inferior overall survival (OS) in a cohort of 362 patients. These factors were incorporated together to develop a prognostic nomogram. The first three variables had a value of 1, and the fourth had a value of 2. Patients with total scores 0, 1, and 2 to 5 were assigned to low-, medium-, and high-risk groups, respectively (12).

### Response Criteria

Hematological complete response (CR_H_) was assessed by immunofixation electrophoresis and free immunoglobulin light-chain negativity in both serum and urine. VEGF complete response (CR_V_) was defined as a decrease in VEGF to a normal concentration (<600 pg/mL). Patients were asymptomatic after finishing therapy, and levothyroxine was tapered off in 1 month before repeating thyroid function test. Thyroid remission is defined as the transfer from hypothyroidism or subclinical hypothyroidism to euthyroidism or from hypothyroidism to subclinical hypothyroidism.

### Statistical Analysis

Progression-free survival (PFS) was the time from treatment to recurrence, the deterioration of clinical symptoms, or death. OS was defined as the time from treatment to death. The date of last follow-up was February 1, 2019. Data analysis was performed with the statistical software package SPSS 22.0 (SPSS, Inc., Chicago, IL, USA). The χ^2^ test, or Fisher exact test when appropriate, was used to determine the significance of differences in the values of categorical variables. Kendall tau analysis was used for ordered categorical variables. Paired *t* test was used to compare numerical variables before and after treatment. Kaplan–Meier method was used to plot survival curves, and differences were compared with the log-rank test. *P* < 0.05 was considered statistically different.

## Results

### Baseline Endocrine Evaluations

Baseline characteristics and endocrine function evaluation are shown in Table 1. The median age at diagnosis was 48 years (range, 21–74 years). One hundred sixty-one were older than 50 years; 62.7% (240/383) was male. The median Overall Neuropathy Limitation Scale (ONLS) score was used to evaluate neurologic disabilities as reported previously (13). The median ONLS score was 4 of a possible 0 to 12 points, and peripheral neuropathy was confirmed by electromyography in the 45 patients with ONLS scores of 0. A total of 61.9% of the patients were positive for immunoglobulin A–type heavy chain monoclonal immunoglobulin. The light chains were λ type in 99.2% (380/383) of patients. Risk stratification resulted in 89 low-risk (23.2%), 147 medium-risk (38.4%), and 147 high-risk (38.4%) of patients.

**Table 1:** Baseline endocrine characteristics of 383 patients.

	***n*(%)**
Age >50 years	161 (42.0)
Male, %	240 (62.7)
Thyroid	
Clinical hypothyroidism	79 (20.6)
Subclinical hypothyroidism	138 (36.0)
Euthyroidism	118 (30.8)
Low FT3 with normal range of FT4 and TSH	43 (11.2)
Elevated TgAb	18 (13.5) (*n* = 133)
Elevated TPOAb	11 (8.3) (*n* = 133)
Bone metabolism	
Elevated β-CTX	257 (92.7) (*n* = 277)
Elevated ALP	23 (13.6) (*n* = 169)
Disease-related bone turnover	128 (47.3) (*n* = 266)
Adrenal gland	
Adrenocortical insufficiency	43 (13.6) (*n* = 316)
Elevated ACTH with normal cortisol levels	222 (70.3) (*n* = 316)
Gonads	
Hyperprolactinemia	207 (62.7) (*n* = 330)
Female	
Hypoestrogenemia	60 (48.0) (*n* = 125)
Hypogonadotropic hypogonadism	18 (30.0) (*n* = 60)
Male	
Hypoandrogenemia	51 (22.6) (*n* = 226)

Similar to Mayo's data (4) and the recent report from Europe (5), thyroid disorders presented in a total of 56.6% of patients, including 20.6% (79/383) of overt hypothyroidism and 36.0% of subclinical hypothyroidism (138/383). A total of 30.8% (118/383) of patients had euthyroidism at baseline. Another 11.2% (43/383) of patients had low serum FT3 levels with normal ranges of FT4 and TSH. Elevated TgAb and TPOAb levels were found in 13.5% (18/133) and 8.3% (11/133) of patients, respectively. Adrenal insufficiency was noted in 13.6% (43/316) of patients, which was lower than that reported from Europe (5). Hyperprolactinemia was found in 62.7% (207/330) of our patients, similar to that reported from Europe (5). The elevated prolactin (PRL) level was at a median of 24.68 ng/mL (range, 13.26–2,300 ng/mL) in male patients and 46.33 ng/mL (range, 30.01–1,377 ng/mL) in female patients. Hypogonadism was noted in 48.0% (60/125) of female and 22.6% (51/226) of male patients, respectively. Elevated β cross-linked C-telopeptide of type 1 collagen (β-CTX) and alkaline phosphatase (ALP) were noted in 92.7% (257/277) and 13.6% (23/169) of patients, respectively. In the present study, a total of 7.3% (26/355) of patients had impaired fasting blood glucose tolerance, and another 9.0% (32/355) of patients had fasting blood glucose equaling to or greater than 7 mmol/L.

### The Status of Thyroid Function Parallels With Baseline Risk Stratification

**Figure 2:**
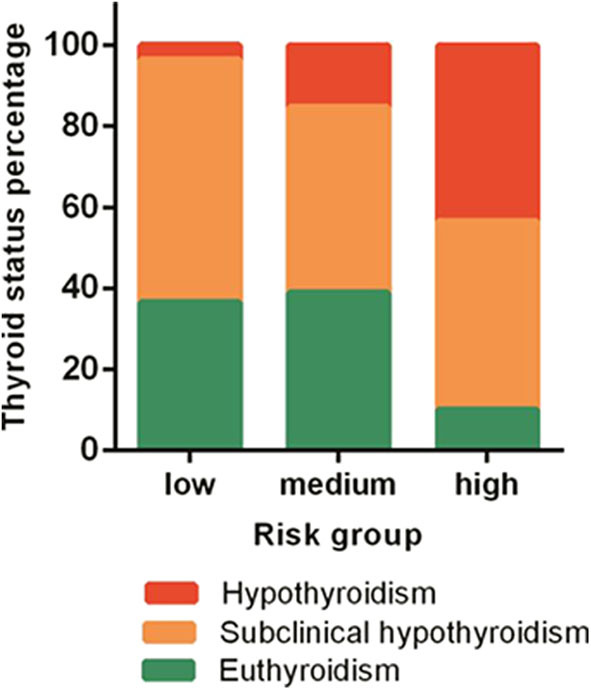
The thyroid status composition in different risk groups.

At baseline, hypothyroidism was noted: 3.4% (3/89), 13.6% (20/147), and 38.1% (56/147) in patients with low-, medium-, and high-risk groups respectively (Figure 2). In the high-risk group, only 10.1% of patients had normal thyroid function. The percentage of normal thyroid function was 43.4 and 46.5% in the low- and medium-risk groups, respectively. Patients' thyroid function was significantly related with their baseline risk stratification (*p* < 0.001).

### Baseline Hypothyroidism Was a Prognostic Factor for OS and PFS

**Figure 3:**
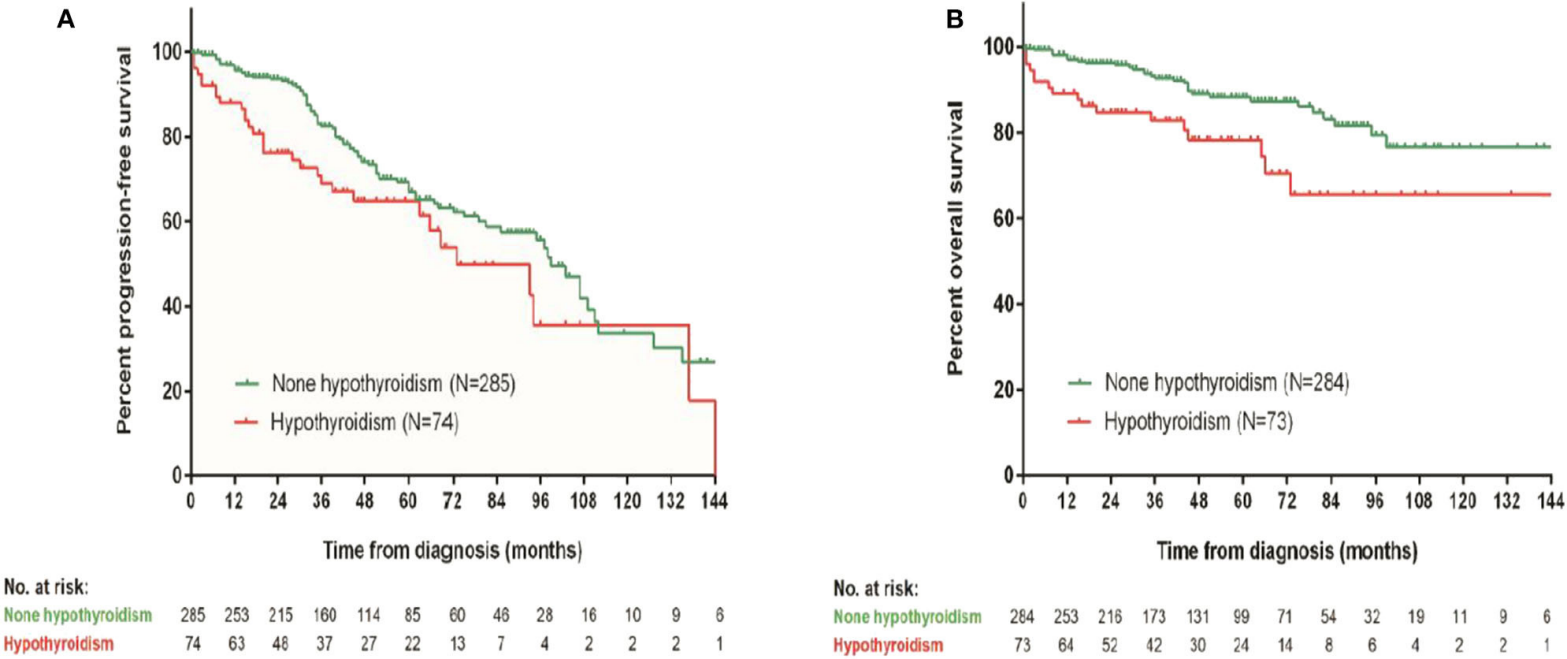
Survival comparison between patients with and without baseline hypothyroidism. **(A)** Progression-free survival, **(B)** overall survival.

Patients with baseline hypothyroidism had a significant inferior PFS (*p* = 0.028) and OS (*p* = 0.006). Three-year PFS rates in patients with and without baseline hypothyroidism were 68.9 vs. 82.5%, respectively. Three-year OS rates in patients with and without baseline hypothyroidism were 82.8 vs. 92.8%, respectively (Figure 3). However, patients with subclinical hypothyroidism at baseline were not significantly different from patients with euthyroid in PFS (*p* = 0.941) or OS (*p* = 0.939). Patients' baseline hypothyroidism was not related with CR_H_ (*p* = 0.410) or CR_V_ (*p* = 0.320) rates.

### Patients Achieved Similar Significant Thyroid Function Improvement After Three Different Regimens

**Figure 4:**
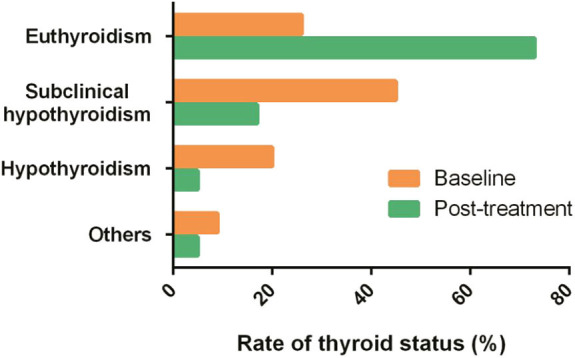
Pretreatment and posttreatment thyroid status composition.

In all these patients treated with three different regimens, hypothyroidism decreased from 20.5 to 5.1% after treatment (Table 2). Percentage of subclinical hypothyroid decreased from 45.0 to 16.8%. Percentage of euthyroidism increased from 26.4 to 73.3% (Figure 4). Thyroid function improvement was then assessed in a total of 181 patients who had abnormal thyroid function at baseline. Patients who achieved thyroid response were 85.7% (18/21), 73.4% (69/94), and 78.8% (52/66) in the MDex, ASCT, and LDex groups, respectively. Thyroid response rate in 6 to 18 months was not significantly different between treatment groups (*p* = 0.429).

**Table 2:** Thyroid function improvement of patients with baseline thyroid abnormalities.

	**Total**			**MDex (*n* = 77)**			**ASCT (*n* = 178)**			**LDex (*n* = 128)**		
	**Pre**	**Post**	***p***	**Pre**	**Post**	***p***	**Pre**	**Post**	***p***	**Pre**	**Post**	***P***
**Thyroid function**	***n*** **=** **17**			***n*** **=** **21**			***n*** **=** **94**			***n*** **=** **66**		
FT3 (pg/mL)	1.91 (0.28–3.69)	2.74 (0.59–4.33)	<0.001	1.85 (0.77–2.92)	2.80 (2.17–3.38)	<0.001	2.03 (0.52–3.69)	2.71 (0.59–4.33)	<0.001	1.76 (0.28–3.19)	2.76 (1.00–4.05)	<0.001
FT4, (ng/dL)	0.93 (0.34–1.48)	1.13 (0.00–2.17)	<0.001	0.84 (0.34–1.48)	1.10 (0.77–1.67)	0.008	0.99 (0.34–1.46)	1.11 (0.00–2.17)	0.001	0.88 (0.38–1.35)	1.18 (0.49–1.75)	<0.001
TSH, (μIU/mL)	9.38 (0.58–36.91)	4.04 (0.032–55.05)	<0.001	9.75 (4.48–22.31)	2.66 (0.60–8.14)	<0.001	8.67 (2.56–36.91)	4.09 (0.03–55.05)	<0.001	10.26 (0.58–36.85)	4.38 (0.25–50.21)	<0.001

### Patients With Thyroid Response Had Better Disease Remission and Survivals

**Figure 5:**
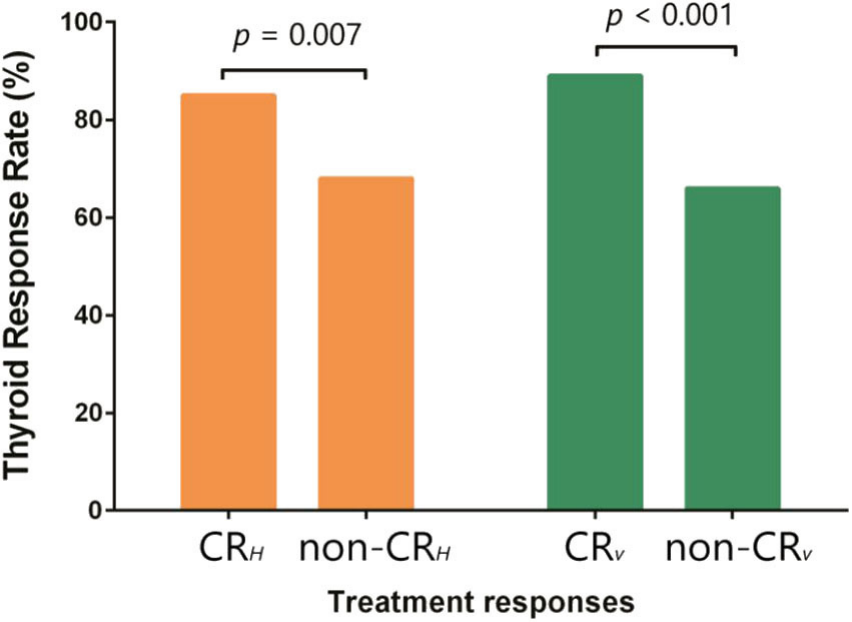
Responses comparison between patients with and without CR_H_ and CR_V_.

**Figure 6:**
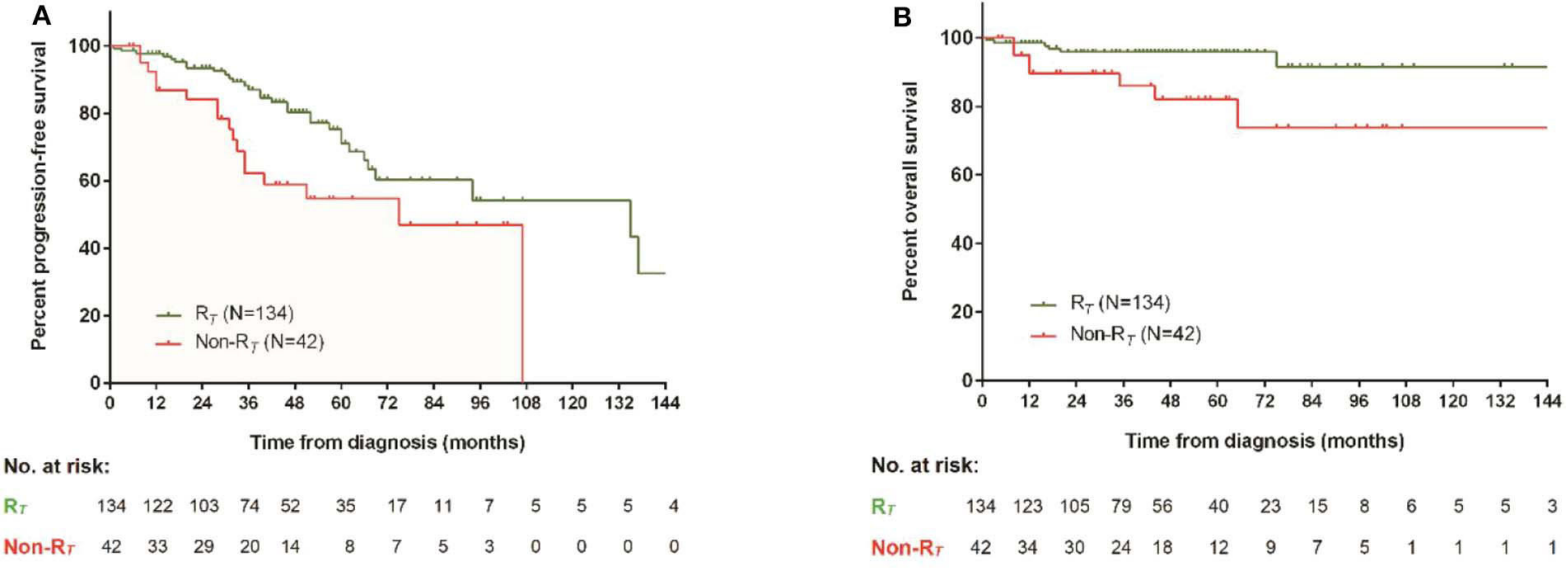
Survival comparison between patients with and without thyroid remission. **(A)** Progression-free survival, **(B)** overall survival.

Patients who achieved CR_H_ had a thyroid response rate of 85.2%, significantly higher than 68.2% of non-CR_H_ patients (*p* = 0.007). Patients with CR_V_ also achieved a higher thyroid response than patients without CR_V_ (89.8 vs. 66.2%, *p* < 0.001) (Figure 5). FT3 and FT4 increased, and TSH reduced significantly in patients with baseline thyroid abnormalities, regardless of the initial treatment (Table 2). Besides, thyroid response could be further translated into better PFS (*p* = 0.010) and OS (*p* = 0.005) (Figure 6).

Disease-related bone turnover was marked by elevated β-CTX or ALP and normal ranges of serum calcium and phosphate. The baseline disease-related bone turnover was not significantly correlated with risk stratification (*p* = 0.550) and also prognostic for neither PFS (*p* = 0.133) nor OS (*p* = 0.176).

## Discussion

In this retrospective study, we evaluated endocrine characteristics and responses to three different regimens in a large cohort of POEMS patients. Our results showed that (1) hypothyroidism, hyperprolactinemia, and hypogonadism are common endocrinopathies in POEMS syndrome; (2) thyroid function was significantly related with baseline risk stratification; (3) thyroid function improvement was significant after treatment, regardless of the initial regimens; (4) patients with baseline hypothyroidism had inferior OS and PFS; (5) the baseline disease-related bone turnover was not significantly correlated with risk stratification and also not prognostic for either PFS or OS.

Endocrine disorders are important but poorly understood characteristics of POEMS syndrome. Clinical spectra of endocrinopathy are similar in different cohorts including our previous work and the present study (4–6), which suggest that hypothyroidism, hyperprolactinemia, and hypogonadism are common endocrinopathies in POEMS syndrome. Pathogenesis of these disorders is unknown so far. Because circulating antibodies directed against specific hormone receptors or hormones *per se* have not been found (14) and no pathological characteristics had been found at autopsy (15), endocrine abnormalities are functional rather than structural disturbances of gland function.

Given the multisystem nature of POEMS syndrome, there is a large group of parameters that should be assessed in POEMS patients. For simplified organ response, evaluations are limited to systems relating with most morbidities, including peripheral neuropathy assessment, extravascular overload, and pulmonary function evaluation. Recommendations about organ response evaluation have been suggested recently (16). There is no consensus about the monitoring and management of endocrine disturbances so far. In Mayo series, adrenal insufficiency and elevated ACTH levels were noted in 67% (6/9) and 48% (13/27) of patients, respectively. In a recent series from Europe, adrenal insufficiency was noted in 17% (10/59) of patients (5). In our present work, adrenal insufficiency was found in 13.6% (43/316) of patients at baseline; 70.3% (222/316) had elevated ACTH with normal cortisol levels in the morning; 70.3% of patients had elevated ACTH with normal cortisol levels, but it was hard to decide whether it was pulsatile secretion or partial adrenal deficiency. Absent or subnormal response to metyrapone or ITT is diagnostic for adrenal insufficiency, whereas corticotropin releasing hormone(CRH) stimulation test is helpful for the differentiation between secondary AI or tertiary AI. But all these provocation tests had not been employed in this study because they are inconvenient in daily work, and at the same time, the LDex and MDex regimens all include dexamethasone.

In our previous work, improvement of sexual function (8) and thyroid function (7) after combination therapy of lenalidomide and dexamethasone was described in patients with newly diagnosed POEMS syndrome. The correlation between VEGF levels and thyroid function improvement had been reported in our previous work (7). Whether these improvements are specific to the combined regimen is an interesting issue to investigate. In this retrospective study, we compared the thyroid function improvement in patients treated with three different regimens including ASCT, MDex, and LDex and found that FT3 and FT4 increased, and TSH reduced significantly in patients with baseline thyroid abnormalities, regardless of the initial treatment. These results further suggest that endocrine abnormalities are functional disturbances, and there is potential common pathogenesis in these endocrinopathies.

In patients with POEMS syndrome, the median survival time is commonly longer than 5 years. The main causes of death include progressive inanition, cardiopulmonary dysfunction, renal failure, and infection (17). The impact of endocrine disorders on OS and PFS is unknown so far. In our previous work, patients with overt hypothyroidism at baseline had an inferior 1-year OS than euthyroidism and subclinical hypothyroidism group (7). In the present work, we further demonstrated that baseline thyroid function was significantly related with baseline risk stratification and was a prognostic factor for OS and PFS. Further evaluation of thyroid function on metabolism and volume overload in POEMS syndrome will provide more information about the significance of thyroid hormones in the progression of disease.

There are some limitations to this study. The first and most important point is the shortness of retrospective single-center study. Another point is that the endocrine system plays pivotal roles in metabolic regulation, but we assessed only bone turnover markers. In our study, elevated β-CTX and ALP were noted in 92.7 and 13.6% of patients, respectively, but baseline disease-related bone turnover was not significantly correlated with risk stratification and was not prognostic for either PFS or OS. Further prospective studies to assess metabolic parameters in POEMS patients will provide more information for this complex syndrome. The third point is that other systems including prolactin, gonadal function, and hypophyseal–pituitary–adrenal axis are worth reporting. But we had not collected relative data because this is a retrospective study. Prospective studies to evaluate the speed of normalization of the endocrine abnormalities are also needed for better clinical management of POEMS syndrome.

In summary, endocrinopathies including hypothyroidism, hyperprolactinemia, and hypogonadism are common endocrinopathies in POEMS syndrome. Thyroid function significantly improved regardless of the initial regimen. Baseline thyroid function parallels with risk stratification and is an independent prognostic factor for OS and PFS. Further studies are essential for efficient monitoring and management of endocrine disturbances in this rare syndrome.

## Data Availability Statement

The raw data supporting the conclusions of this article are available from authors upon request.

## Ethics Statement

The studies involving human participants were reviewed and approved by Institutional Review Board of Peking Union Medical College Hospital. Written informed consent for participation was not required for this study in accordance with the national legislation and the institutional requirements.

## Author Contributions

HY performed data analysis and manuscript composition. HZ collected data and performed statistical analysis. XG and XH contributed in data collecting. XC and DZ contributed in clinical research. JL designed the research study. All authors contributed to the article and approved the submitted version.

## Conflict of Interest

The authors declare that the research was conducted in the absence of any commercial or financial relationships that could be construed as a potential conflict of interest.
